# Worldwide impacts of landscape anthropization on mosquito abundance and diversity: A meta‐analysis

**DOI:** 10.1111/gcb.16406

**Published:** 2022-09-27

**Authors:** Antoine Perrin, Olivier Glaizot, Philippe Christe

**Affiliations:** ^1^ Department of Ecology and Evolution University of Lausanne Lausanne Switzerland; ^2^ Museum of Zoology Lausanne Switzerland

**Keywords:** agricultural development, deforestation, Diptera, landscape changes, pathogen vectors, urbanization

## Abstract

In recent decades, the emergence and resurgence of vector‐borne diseases have been well documented worldwide, especially in tropical regions where protection and defense tools for human populations are still very limited. In this context, the dynamics of pathogens are influenced by landscape anthropization (i.e., urbanization, deforestation, and agricultural development), and one of the mechanisms through which this occurs is a change in the abundance and/or diversity of the vectors. An increasing number of empirical studies have described heterogeneous effects of landscape anthropization on vector communities; therefore, it is difficult to have an overall picture of these effects on a global scale. Here, we performed a meta‐analysis to quantify the impacts of landscape anthropization on a global scale on the presence/abundance and diversity of mosquitoes, the most important arthropods affecting human health. We obtained 338 effect sizes on 132 mosquito species, compiled from 107 studies in 52 countries that covered almost every part of the world. The results of the meta‐analysis showed an overall decline of mosquito presence/abundance and diversity in response to urbanization, deforestation, and agricultural development, except for a few mosquito species that have been able to exploit landscape anthropization well. Our results highlighted that these few favored mosquito species are those of global concern. They, thus, provide a better understanding of the overall effect of landscape anthropization on vector communities and, more importantly, suggest a greater risk of emergence and transmission of vector‐borne diseases in human‐modified landscapes.

## Graphical abstract

Understanding how land‐use changes affect mosquito abundance and diversity is a step towards a better understanding of vector‐borne pathogen dynamics in human‐modified landscapes. In this meta‐analysis, we highlight that urbanization, deforestation, and agricultural development lead to a general decline in mosquito diversity and a decrease in most mosquito abundance, favouring the only species adapted to human‐modified landscapes. These few species are confirmed vectors of multiple human pathogens. This thus suggests a higher human risk of vector‐borne diseases in more anthropized areas.

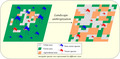

## 1 INTRODUCTION

Vector‐borne diseases (VBDs) have been a major human health problem in recent decades. Indeed, more than 80% of the world population lives in areas exposed to at least one vector‐borne pathogen, and almost all VBDs occur in the tropics where access to medical care, safe drinking water, and sanitation systems is still not guaranteed (Golding et al., 2015; WHO, 2014). In addition, an increased frequency of epidemic transmission and an expanding geographic distribution have been observed for many VBDs (Gubler, 2009; Müller et al., 2019). For instance, the incidence of dengue has grown dramatically around the world, with a 30‐fold increase over the last 50 years. Several major outbreaks of chikungunya have occurred in several places around the world in the last decade, and a resurgence of yellow fever has been documented after years of decline in both Africa and South America (Gardner & Ryman, 2010; WHO, 2014). Therefore, there is an urgent need to understand the global drivers of vector‐borne pathogen dynamics to better predict, diagnose, monitor, and control future pandemic outbreaks.

One of the main identified drivers of disease emergence is the anthropization of the landscape (Despommier et al., 2007; Gibb et al., 2020; Morand & Lajaunie, 2018; Patz et al., 2000). Landscape anthropization can be defined through three main environmental components: urbanization, deforestation, and agricultural development. Although these three components have implications for the emergence and proliferation of VBDs (Gubler, 1998; Vora, 2008), they are closely related; one can be the cause or the consequence of the others (DeFries et al., 2010; Nathaniel & Bekun, 2020; Tilman et al., 2001). Despite the complex and variable effects of landscape anthropization on pathogen dynamics, several systematic reviews have highlighted that an increase in pathogen transmission and prevalence was usually associated with urbanization, deforestation, and agricultural development (Brearley et al., 2013; Gottdenker et al., 2014; White, 2015), but the mechanisms behind these landscape anthropization effects remain to be investigated.

There is a wide variety of mechanisms in action considering the inherent complexity of the spread of VBDs since it involves at least three organisms, namely, a parasite, a vector, and a host. Endoparasites are not directly exposed to landscape changes during their life cycle; these changes can therefore only act on these parasites indirectly through their effects on the vector and/or the host (Ferraguti et al., 2018). In this context, the expansion of the vector in human‐modified landscapes has led to the emergence of several diseases caused by parasites in humans (Estrada‐Peña et al., 2014; Morand & Lajaunie, 2018). This is, for example, the case for Chagas disease, transmitted by triatomine bugs; trypanosomiasis, transmitted by tsetse flies (*Glossina* species); filariasis, transmitted by phlebotomine sandflies; bilharzia, transmitted by freshwater snails; onchocerciasis, transmitted by black flies; and malaria, dengue, Rift Valley fever, and West Nile fever transmitted by mosquitoes (Morand & Lajaunie, 2018).

During the past century, it has become established that mosquitoes are the most important arthropods affecting human health (Foster & Walker, 2019) and are the most widely studied taxa among invertebrates with medical importance, given their role as vectors of many pathogens (Chaves, 2017). Mosquitoes are found on every continent except Antarctica and hundreds of millions of dollars are spent annually to protect humans from mosquito bites all over the world (Diagne et al., 2020; Foster & Walker, 2019). Moreover, through a modification of mosquito abundance and diversity, landscape anthropization has led to a change in the prevalence of parasites responsible for avian malaria in Spain and Cameroon (Ferraguti et al., 2016, 2018; Tchoumbou et al., 2020). Numerous empirical studies have examined the effects of landscape anthropization on mosquito communities, and some authors have concluded that we can already draw general patterns. Overall, it has been suggested that mosquito abundance and diversity are higher in natural and rural areas than in urban areas (e.g., Ferraguti et al., 2020). However, no quantitative review on the subject exists in the literature except on a particular mosquito genus and/or a particular relationship [e.g., land cover and *Aedes* presence (Sallam et al., 2017), deforestation and mosquito abundance (Burkett‐Cadena & Vittor, 2018)].

Here, we conducted a comprehensive research review and a meta‐analysis of the existing literature to highlight the overall impact of landscape anthropization on mosquito presence/abundance and diversity as a step towards a better understanding of vector‐borne pathogen dynamics in human‐modified landscapes. We considered all available studies, whether they used a spatial approach (e.g., data that compared several rural and urban sites at a specific time), a temporal approach (e.g., data that compared one rural site and one urban site across time), or both. We excluded studies that did not simultaneously sample disturbed and undisturbed sites since mosquito populations could vary significantly from year to year (Chase & Knight, 2003; Reisen et al., 2008; Wolda & Galindo, 1981). We pooled the effects of the three environmental components (i.e., urbanization, deforestation, and agricultural development) to obtain the largest picture of the impacts of landscape anthropization and the greatest number of effect sizes. The specific objectives of this meta‐analysis were (i) to quantitatively test the prediction of a decrease in mosquito abundance and diversity in human‐modified landscapes on a global scale; (ii) to investigate how different mosquito species respond to the three environmental components; and (iii) to assess whether the response is linked to the ability to transmit human pathogens of mosquito species.

## 2 MATERIALS AND METHODS

### 2.1 Literature search

**FIGURE 1:**
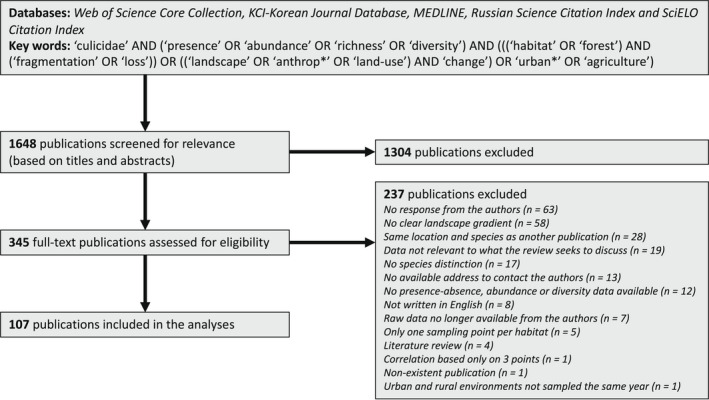
PRISMA flow diagram (Liberati et al., 2009) used for this meta‐analysis on the effects of landscape anthropization on mosquito presence/abundance and diversity.

Peer‐reviewed publications were sourced from the following databases: *Web of Science Core Collection*, *KCI‐Korean Journal Database*, *MEDLINE*, *Russian Science Citation Index* and *SciELO Citation Index* (http://www.webofknowledge.com) using a combination of keywords including Culicidae, presence, abundance, richness, diversity, habitat loss, fragmentation, anthropogenic, landscape/land‐use change, urban, agriculture and forest (Figure 1). The search generated 1648 studies published until June 2021.

We first eliminated the references that did not fit the purpose of our review based on their title and abstract. Then, we excluded studies whose objectives were not to test the effect of landscape anthropization on mosquito abundance and diversity after a full reading of the text. In addition, we excluded studies that did not fulfil the following eligibility criteria: the study (i) was written in English; (ii) identified mosquitoes to species, (iii) estimated mosquito presence/absence, abundance, or diversity; (iv) used a clear landscape anthropization gradient; (v) used data sampled the same year in each environment; (vi) had at least two sampling points per habitat or more than three sampling points on a landscape anthropization gradient; (vii) was not a literature review; and (viii) had available raw data. This resulted in 107 studies from which data were extracted. The process and outcome of the literature search were summarized in the Preferred Reporting Items for Systematic Reviews and Meta‐Analyses (PRISMA) flow diagram (Figure 1), as well as in the supporting information (Appendix S1).

### 2.2 Data extraction and effect size calculation

For each study, we recorded the response variable studied [mosquito presence/abundance or diversity (i.e., species richness, Simpson or Shannon index) depending on the study], the mosquito taxonomy (genus and species), and the stage (immature or adult). We extracted the correlation coefficient between the gradient of landscape anthropization (i.e., deforestation, agricultural development, or urbanization) and the response variable from text, tables, or figures (with the “digitize” R package; Poisot, 2011) within publications, supplementary materials, or solicited authors. For studies that made comparisons of mosquito abundance or diversity between two habitat categories (e.g., urban vs. rural), we extracted the means and standard deviations. Finally, when proportions of individuals were given (i.e., presence/absence between two habitat categories), we used the odds ratio (Cooper et al., 2019).

As not all studies reported the same effect size metrics, their direct comparison was not possible. We, thus, used conversions from Cooper et al. (2019) and Harrer et al. (2021) to obtain the correlation coefficient *r*, which is a common metric of effect size allowing comparison between studies. To comply with the application conditions of meta‐analytical tests (e.g., the distribution normality of effect sizes), we then converted each *r* into Fisher's *Zr* (Cooper et al., 2019). The transformation from *r* to *Zr* is given by *Zr* = 0.5 × ln((1 + *r*)/(1 − *r*)). After the analyses, meta‐analytic *Zr* means were back transformed into meta‐analytic *r* means to facilitate interpretations.

### 2.3 Meta‐analyses

**FIGURE 2:**
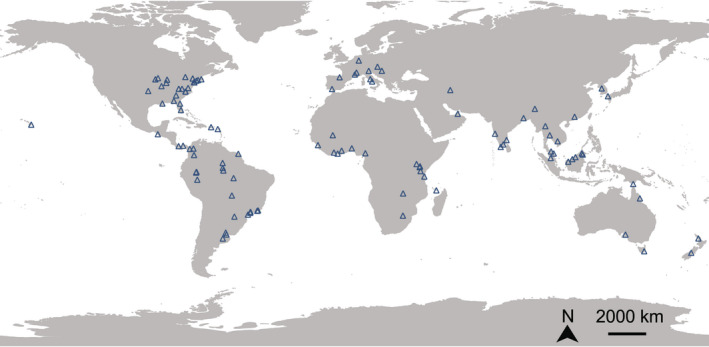
Geographic distribution of the 107 studies used in the meta‐analysis (i.e., blue triangles) testing the effects of landscape anthropization on mosquito presence/abundance and diversity.

We tested the overall effect of landscape anthropization on mosquito diversity (hereafter called *meta‐diversity* analysis) using a random‐effects model to estimate the mean of the distribution of effect sizes. Effect sizes (*Zr*) were used as the dependent variables, and their variance was calculated using the formula: 1/(*n* − 3) (Cooper et al., 2019), where *n* is the sample size associated with each effect size. Sample sizes were determined from the number of sampling sessions for studies that used a temporal approach and from the number of sampling sites for studies that used a spatial approach. For the overall effect of landscape anthropization on mosquito presence/abundance (hereafter called *meta‐abundance* analysis), we ran a multilevel model to consider several types of nonindependence in the data arising from multiple effect sizes originating from the same study or the same species (Figure 2; Appendix S1). We, thus, accounted for species‐ and study‐level nonindependence by including mosquito species and study ID as random factors in the model. Meta‐analytic means and their confidence intervals were obtained for the *meta‐diversity* and *meta‐abundance* analyses by the intercept test of the random‐effects model and the multilevel model, respectively.

We tested the random factor “species” with a model comparison and a likelihood ratio test (LRT). We also assessed the inconsistency in effect sizes among studies by computing *I* ^*2*^, which quantifies the percentage of variability in the effect sizes that is not due to sampling error. In the case of multilevel models, we partitioned *I* ^*2*^ between the two random factors (i.e., study and mosquito species factors). According to Higgins et al. (2003), heterogeneity was considered low, moderate, and high when *I* ^*2*^ = 0.25, 0.50, and 0.75, respectively.

### 2.4 Mixed‐effects meta‐regression analyses

After estimating the overall effect of landscape anthropization on mosquito presence/abundance, we ran a meta‐regression to assess the contribution of one moderator to the heterogeneity of effect sizes. As in multilevel models, mosquito species and study ID were entered as random factors within all models. We identified the mosquito's ability to transmit human pathogens as a moderator that could explain the heterogeneity of the landscape anthropization effects on mosquito presence/abundance. Indeed, Burkett‐Cadena and Vittor (2018) systematically reviewed the literature focusing on mosquito abundance changes between forested and deforested areas and showed that vectors of human pathogens are more abundant in deforested areas, while a reverse trend was observed for non‐vectors. We, thus, tested whether this pattern is still observed with a global view of the landscape anthropization effects on mosquitoes and with a much larger number of publications (Burkett‐Cadena and Vittor (2018): *N* = 17 publications, and neither a meta‐analytic approach was used nor were meta‐analytic means provided).

According to Becker et al. (2020) and Wilkerson et al. (2021), we identified 14 of the most important VBDs for humans (i.e., malaria, chikungunya, Ross River fever, equine encephalomyelitis, O'nyong‐nyong, Sindbis fever, yellow fever, dengue, Zika virus disease, West Nile fever, Japanese encephalitis, Usutu virus disease, Rift Valley fever, and lymphatic filariasis) and identified the number of these 14 VBDs that were associated with each mosquito species. We considered that the number of VBDs associated with a mosquito species reflected its ability to transmit vector‐borne pathogens. To reduce the number of categories in our models, we ranged mosquito species into five arbitrary classes of associated VBD numbers (0, 1 to 3, 4 to 6, 7 to 9, and 10 or more associated VBDs).

As there is an advantage for mosquito species that feed on mammals and more specifically on humans in anthropized environments (due to higher human density) compared to other mosquito species, we planned to add a feeding pattern moderator in the meta‐regression models. We hypothesized that mosquito species associated with mammals would be positively affected by landscape anthropization while mosquito species associated with birds, amphibians and more generally wildlife would be negatively affected. However, almost all mosquito species studied in this study feed on mammals and there was therefore not enough variability in the feeding preference (Becker et al., 2020; Wilkerson et al., 2021) to test this hypothesis.

Finally, we did not test a moderator representing the type of disturbance (i.e., urbanization, deforestation, and agricultural development) due to the strong correlations between these three environmental components.

### 2.5 Publication bias

Publication bias occurs when the publication of studies depends on their results (Rothstein et al., 2005). This is especially true for small studies where only very large effects become significant. This publication bias can lead to overestimating or underestimating the overall effect size according to a theoretical expectation that could be invalid (Harrer et al., 2021). We quantified the publication bias across both *meta‐diversity* and *meta‐abundance* analyses using both Egger's regression (Egger et al., 1997) and Duval & Tweedie trim‐and‐fill (Duval & Tweedie, 2000) methods (i.e., two publication bias analyses *per* response variables for a total of four analyses). Following the recommendation of Nakagawa and Santos (2012), we conducted Egger's regression and trim‐and‐fill methods on the residuals for the *meta‐abundance* analysis because they account for nonindependence due to multiple effect sizes originating from the same study or the same species.

All calculations were performed with the *metafor* (Viechtbauer, 2010) and *meta* (Balduzzi et al., 2019) packages available in R software (version 4.1.1; R Core Team, 2021).

## 3 RESULTS

### 3.1 Summary of the literature review

The 107 studies were published between 1992 and 2021, covered 52 countries distributed over five continents, with 16, 21, 10, 6, 28, and 26 publications from Africa, Asia, Europe, Oceania, North America, and South America, respectively (Figure 2). The full data set comprises 338 effect sizes, including 132 mosquito species, with 29 effect sizes obtained from 29 studies for the *meta‐diversity* analysis and 309 effect sizes obtained from 98 studies for the *meta‐abundance* analysis. The three main landscape anthropization gradients were studied in the literature (i.e., urbanization, deforestation, and agricultural development), but most studies were focused on urbanization effects (70% of studies; Appendix S1).

Seventy‐one mosquito species studied were mammophilic or opportunistic, while only two species were ornithophilic and one species was associated with amphibians. To our knowledge, the feeding preference of the remaining 58 species is unknown. In addition, the most studied mosquitoes in a landscape anthropization context were *Aedes albopictus* (36 studies), *Aedes aegypti* (25 studies), *Culex pipiens* (20 studies), *Culex quinquefasciatus* (15 studies), and *Aedes vexans* (11 studies), all of which were opportunistic or had a feeding preference associated with mammals (Appendix S1).

### 3.2 Overall landscape anthropization effects on mosquito presence/abundance and diversity

**FIGURE 3:**
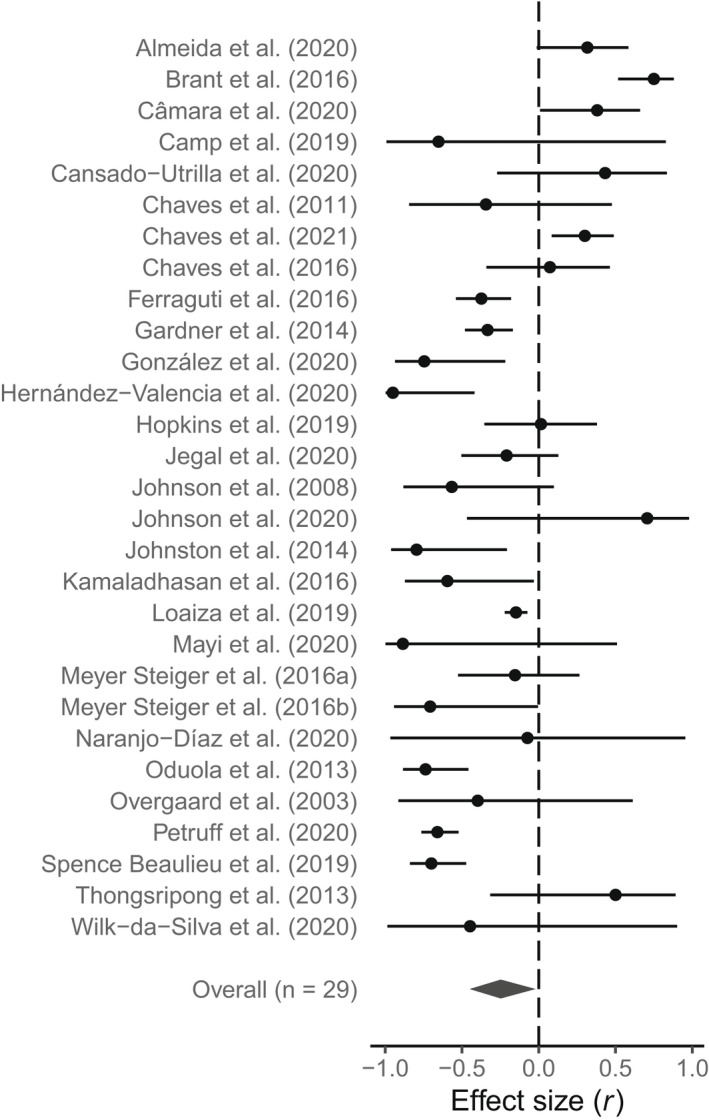
Effect size of landscape anthropization on mosquito diversity for each study and meta‐analytic mean (*overall*) based on the correlation coefficient (±95% CI).

**FIGURE 4:**
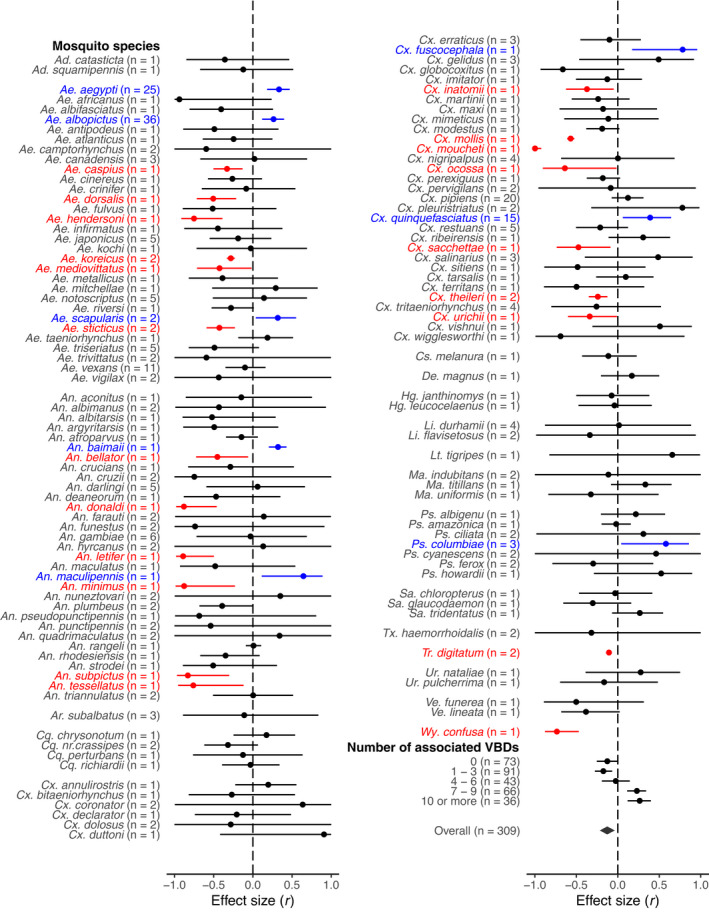
Meta‐analytic mean per mosquito species and class of associated VBD (Vector‐borne disease) number as well as meta‐analytic mean (*overall*) based on the correlation coefficient (±95% CI) for the landscape anthropization effects on mosquito presence/abundance. n refers to the number of effect sizes. The colours blue, red, or grey, respectively, showed whether mosquito species was positively, negatively, or not affected by landscape anthropization. *Ad*. = *Aedeomyia*, *Ae*. = *Aedes*, *An*. = *Anopheles*, *Ar*. = *Armigeres*, *Cq*. = *Coquillettidia*, *Cx*. = *Culex*, *Cs*. = *Culiseta*, *De*. = *Deinocerites*, *Hg*. = *Haemagogus*, *Li*. = *Limatus*, *Lt*. = *Lutzia*, *Ma*. = *Mansonia*, *Ps*. = *Psorophora*, *Sa*. = *Sabethes*, *Tx*. = *Toxorhynchites*, *Tr*. = *Trichoprosopon*, *Ur*. = *Uranotaenia*, *Ve*. = *Verrallina*, *Wy*. = *Wyeomyia*.

From the global data set, there was a significant negative overall effect size of landscape anthropization on both mosquito diversity (*r* = −0.25, 95% CI: −0.45 to −0.02, *p* = .03; Figure 3) and mosquito presence/abundance (*r* = −0.13, 95% CI: −0.22 to −0.04, *p* = .006; Figure 4). Overall, we found substantial heterogeneity not caused by sampling error in the *meta‐diversity* analysis (*I* ^*2*^ = 83%) and in the *meta‐abundance* analysis (*I* ^*2*^ = 96%). More precisely, based on Higgins and Thompson's “rule of thumb” (Higgins et al., 2003), within‐study variations explained a high amount of heterogeneity (*I* ^*2*^ = 53%), whereas between‐study variations and mosquito species variations explained a low amount of heterogeneity (*I* ^*2*^ = 16% and *I* ^*2*^ = 27%, respectively) in effect sizes for the *meta‐abundance* analysis.

The life stage (i.e., adult, immature or both) or type of response (i.e., presence or abundance) did not change the results when they were added to the models (*F*_2,306_ = 2.11, *p* = .12 and *F*_1,307_ = 0.30, *p* = .58, respectively), indicating that these moderators did not explain the observed heterogeneity. Therefore, they were not considered further.

### 3.3 Are landscape anthropization effects associated with mosquito species or the mosquito's ability to transit vector‐borne pathogens?

Overall, we found a significant difference among mosquito species regarding the landscape anthropization effects on mosquito presence/abundance (LRT = 32.3, *p* < .0001; AICc_*full*_ = 500, AICc_*reduced*_ = 530). Eight mosquito species had an increased abundance in response to landscape anthropization, while the others had a decreased abundance or were not affected by urbanization, deforestation, and agricultural development (Figure 4). Moreover, we found a significant association between the landscape anthropization effects on mosquito presence/abundance and the mosquito's ability to transmit vector‐borne pathogens (*F*_4,304_ = 4.27, *p* = .002). Landscape anthropization led to a decrease in the presence/abundance of mosquito species associated with any or a few VBDs, while it led to an increase in the presence/abundance of mosquito species associated with many VBDs (Figure 4).

### 3.4 Publication bias

Based on Egger's regression (Egger et al., 1997), there was no significant evidence for publication bias for either the *meta‐diversity* or the *meta‐abundance* analyses (intercept = −0.33, 95% CI: −1.59 to 0.92, and intercept = −0.28, 95% CI: −0.88 to 0.33, respectively). The trim‐and‐fill analysis estimated a total of 4 and 27 effect sizes missing from the right side of the distribution for the *meta‐diversity* and *meta‐abundance* analyses, respectively. In addition, the correction suggested by this method reduced both the overall effect size for the *meta‐diversity* and the *meta‐abundance* analyses (*r* = −0.16, 95% CI: −0.37 to 0.07 and *r* = −0.05, 95% CI: −0.14 to 0.04, respectively). However, as the trim‐and‐fill method can underestimate the true overall effect size when there is no publication bias and significant heterogeneity among effect sizes (Peters et al., 2007), all publication bias analyses did not suggest evidence of a large publication bias in our data.

## 4 DISCUSSION

The effects of urbanization, deforestation, and agricultural development on mosquito abundance and diversity have been studied in almost every part of the world, with data mainly focused on mosquito species of importance to human health. Overall, the abundance and diversity of mosquitoes are lower in anthropized areas than in natural areas, although not all species responded similarly. While most mosquito species had an abundance that decreased with urbanization, deforestation and agricultural development, the abundance of mosquitoes that are of global concern increased in human‐modified landscapes.

Several comprehensive reviews on the effect of land‐use changes on mosquito ecology identified different trends (Brugueras et al., 2020; Burkett‐Cadena & Vittor, 2018; Madzokere et al., 2020; Sallam et al., 2017; Walsh et al., 1993). First, different mosquito species were affected in different ways by deforestation (Sallam et al., 2017; Walsh et al., 1993), resulting in some cases a decrease but in most cases an increase in infection risk for humans. The underlying mechanisms could be a change in mosquito behaviors such as mating, feeding, and oviposition in anthropized environments (Madzokere et al., 2020; Walsh et al., 1993). Second, the species favored by deforestation are mainly the medically important species, which, thus, leads to an increase in disease risk in deforested areas (Burkett‐Cadena & Vittor, 2018; Madzokere et al., 2020). However, all these comprehensive reviews did not have a meta‐analytical approach and it is difficult to conclude the overall impact of landscape anthropization on mosquito abundance and diversity. Our quantitative synthesis provides meta‐analytic means of the impacts of landscape anthropization on mosquito communities, and our results showed that overall, mosquito abundance and diversity were more often reduced than increased in human‐modified landscapes. These results are in line with other studies that showed that urbanization, deforestation, or agricultural development cause disturbances that affect ecological communities, often leading to an increase in the abundance of a small group of species and a general loss of biodiversity (Fahrig, 2003; McKinney, 2008; Miller & Kauffman, 1998; Newbold et al., 2016).

As suggested by many authors, these results could be explained by the reduction in the availability of breeding areas in urban environments, which led to a lower diversity and a lower surface of wetlands (e.g., lower number of tree holes, ditches, vernal pools, and leaf axils) for mosquitoes (Ferraguti et al., 2016; Gardner et al., 2014; Loaiza et al., 2019; Meyer Steiger et al., 2016a). In human‐modified landscapes, natural environments (e.g., standing water or vegetation) are often replaced with artificial elements for human needs (e.g., housing, shopping centres, and industries). This reduces mosquito abundance and diversity, except for those species capable of growing in artificial and/or temporary ponds (i.e., buckets, ornamental bromeliads, or flowerpots), such as *Ae. albopictus* or *Ae. aegypti* (Wilke et al., 2019). Moreover, in human‐modified landscapes, the blood and sugar sources for adult mosquitoes are lower and less diverse than in natural habitats, especially in forested areas (Gardner et al., 2014). Indeed, the forest habitat has the highest levels of terrestrial species diversity, and almost all taxonomic groups are slightly more likely to occur with increasing forest cover (Newbold et al., 2014). In addition, Aronson et al. (2014) showed that urbanization led to lower densities of both animal and plant species on a global scale. The preference of mosquitoes for different types of habitats could also contribute to the low mosquito abundance and diversity in human‐modified landscapes because several studies showed mosquito species‐specific preferences for understory vegetation or tree cavities more frequently found in natural environments (Burkett‐Cadena et al., 2008; Burkett‐Cadena et al., 2013). However, mosquito resting site preference and selection are not yet fully understood and the underlying mechanisms remain to be determined. Another potential driver of the decrease in mosquito abundance and diversity in human‐modified landscapes is the implementation of mosquito controls in some urban areas to protect human populations. For example, Ferraguti et al. (2016) mentioned that larvicide treatments with *Bacillus thuringiensis* were carried out in some of the studied urban areas and may have reduced the mosquito populations both in terms of density and diversity.

Despite this overall pattern of a decrease in mosquito abundance in response to landscape anthropization, not all mosquito species responded in the same way. First, we found a large heterogeneity among effect sizes, even within a genus or within the same study. These results are not surprising given the variety of mosquito ecological characteristics, such as the difference in dispersal capacities (Verdonschot & Besse‐Lototskaya, 2014), feeding behavior (Becker et al., 2020), larval habitat preference (Almeida et al., 2020), or development time (Russell, 1999). In addition, the predation pressure on mosquitoes in urban areas is lower than that in rural areas (Carlson et al., 2004), which reduces the mechanism of predator‐mediated coexistence and allows mosquitoes that are adapted to human‐modified landscapes to proliferate at the expense of other species (Kesavaraju et al., 2008). Second, we also found an inconsistency in effect sizes within studies even when controlling for heterogeneity due to species identity, which could reflect that the abundance of a species partly depends on other species present in the community. As some species tolerate human‐modified environments, they outweigh other less tolerant species that are then excluded by competitive exclusion. This is in accordance with Johnson et al. (2008) and Lounibos and Juliano (2018) who suggested that competition among mosquito species can be an important factor in determining mosquito abundance, realized niche and future distribution.

The heterogeneity among the effects of landscape anthropization on mosquito abundance is reduced when the ability of mosquitoes to be a vector of human diseases is considered. Indeed, the abundance of mosquitoes that are of global concern increased with urbanization, deforestation, and agricultural development, while the abundance of the others decreased. These results may be due to covariance between life‐history traits and the human disturbance tolerance of species. Species with a large home range, fast growth, and early reproduction are less prone to elimination after a disturbance (Ewers & Didham, 2006; Joseph et al., 2013; Newbold et al., 2018; Purvis et al., 2000) but, at the same time, they are the most competent species for a pathogen (Johnson et al., 2012; Joseph et al., 2013; Lee et al., 2008). In other words, considering life‐history trade‐offs, tolerant species to landscape anthropization may have rapid growth and high reproductive output at the expense of effective pathogen defenses. As suggested by Burkett‐Cadena and Vittor (2018), these results could also be the consequence of a spatial convergence of the pathogen, the host, and the vector through evolutionary processes. Resilient species in human‐modified landscapes may become efficient vectors of pathogens because natural selection may favor the evolution of pathogens infecting the most abundant vector, thus allowing efficient dispersion. Consequently, the most efficient vectors for dispersing human diseases seem to be the species that have a better fitness when humans are present in high density (i.e., in human‐modified landscapes).

Our results have several ecological consequences. First, they suggest an overall loss of biodiversity and a biotic homogenization in human‐modified landscapes. This is in accordance with McKinney (2006), who showed that landscape anthropization was responsible for the homogenization of the environment. In fact, the habitat diversity for flora and fauna in an urban area is much less diversified than that in the same area in a natural environment. Likewise, the urban habitats of two distant cities (e.g., on two different continents) are very similar compared with two adjacent natural habitats in these two cities. This homogenization process in human‐modified landscapes leads to a reduction in the species richness of several taxa, including mammals, birds, reptiles, amphibians, invertebrates, and plants (Chace & Walsh, 2006; Collinge, 2009; McKinney, 2008), and thus to the biological uniqueness of local ecosystems (McKinney & Lockwood, 1999). Second, the increase in abundance of the most efficient vectors for dispersing pathogens, as well as the proximity of humans and vectors in human‐modified landscapes, increases the probability of an encounter between a pathogen and its vector and its transmission to the host. This ultimately makes human‐pathogen interactions more likely in human‐modified landscapes. Given the emergence and re‐emergence of VBDs around the world, it is important to note that landscape anthropization is a factor that allows vectors that are of global concern to thrive.

This meta‐analysis highlights several ways to guide future research. First, the availability of raw data should be increased in empirical studies, giving clear observed effect sizes rather than statistical measures. This would avoid excluding many studies (e.g., 83 in this meta‐analysis) because the data that allow the calculation of effect sizes are not provided in the publication and are not or no longer available from the authors. Second, most studies have focused on mammophilic mosquito species. There are at least two reasons for this: (i) these species are important to human health and are, thus, of primary interest to the medical community and (ii) the mosquito sampling strategy often used in the literature is human landing catches, which is the most accurate and unbiased method to evaluate exposure to mosquito bites or VBDs in humans (Wotodjo et al., 2015). Extending the study of the effects of landscape anthropization to other mosquitoes would provide valuable information on the epidemiological risks to livestock and wildlife in human‐modified landscapes. Third, most studies have been based on a one‐dimensional comparison between disturbed and undisturbed sites without incorporating an explicit quantitative approach to landscape anthropization effects. Therefore, this did not allow us to study the nonlinearity of the relationship between mosquito variables and landscape anthropization or the presence of thresholds, which is important information for the management and conservation of natural environments.

Specific effects associated with each landscape anthropization components on mosquito species have been documented in the literature and reviewed (e.g., Norris, 2004; Vora, 2008). First, deforestation favored mosquitoes with higher vectorial capacities. Hendy et al. (2020) have shown that disease vector species such as *Ae. albopictus* and *Ae. aegypti* was only found within 100 m from the forest edge, while non‐vector and forest specialist species were detected in low numbers within this area. Second, urbanization created many breeding sites and refugia for species capable of using them, as well as a stable source of water during the dry season due to pipes underneath the streets. For instance, *Cx. quinquefasciatus* and *Ae. aegypti* breed most successfully in fresh water‐filled man‐made containers and are therefore found primarily around houses in urban environments (Valentine et al., 2020). Third, agricultural development led to ideal local environments (e.g., higher sedimentation, shallowest water depth) and climate (e.g., warmer temperature) for several mosquito species, including *Ae. albopicus* or *Cx. quinquefasciatus* (Buckner et al., 2011). Here, we were not able to separate the effects of urbanization, deforestation, and agricultural development, although it is essential information in landscape planning. There are two reasons for this: (i) most studies only focused on one gradient without taking into account the others and (ii) the strong correlation between landscape anthropization gradients makes it difficult to quantify their relative effects (e.g., the comparison of forest and urban environments corresponding to both deforestation and urbanization).

## 5 CONCLUSIONS

Our comprehensive review revealed that urbanization, deforestation, and agricultural development have negative impacts on mosquito abundance and diversity on a global scale. However, we found heterogeneity in these overall patterns, with a large difference in response to landscape anthropization among mosquito species. From an ecological point of view, landscape anthropization leads to a general decline in mosquito diversity by reducing most mosquito abundance and by favoring a few species adapted to human‐modified landscapes. These few mosquito species do not belong to the same genus, and a large variation in response is observed among several mosquito species within a genus. This finding indicates that grouping species in genera may not be appropriate for studying the effects of landscape anthropization because the ability to develop and survive in human‐modified landscapes could be different even for two phylogenetically closely related mosquito species. Taking into consideration the ability of a mosquito species to disperse VBDs allowed us to partly explain the heterogeneity of effect sizes. The abundance of mosquitoes of global concern increased in human‐modified landscapes, while the abundance of others decreased. This meta‐analysis revealed a factor that allows vectors of human diseases to thrive, highlighting a positive correlation between the abundance of these vectors and landscape anthropization. This suggests a greater risk of pathogen spillover in human‐modified landscapes and given the rapid land use changes for the benefit of humans, it is important to take this result into account in land‐use planning to reduce the probability of VBD emergence.

## AUTHOR CONTRIBUTIONS

Antoine Perrin, Olivier Glaizot, and Philippe Christe conceived the study. Antoine Perrin developed the methods, screened studies, extracted data, performed the meta‐analysis, and wrote the first draft of the manuscript. All authors contributed to data interpretation, improved the drafts, and approved the final version.

## CONFLICT OF INTEREST

The authors declare that they have no conflict of interest.

## Supporting information


Appendix S1.

## Data Availability

Data and R script for the meta‐analysis are available in Dryad Digital Repository (DOI: 10.5061/dryad.bcc2fqzfm).
